# Comparable High Rates of Extended-Spectrum-Beta-Lactamase-Producing *Escherichia coli* in Birds of Prey from Germany and Mongolia

**DOI:** 10.1371/journal.pone.0053039

**Published:** 2012-12-31

**Authors:** Sebastian Guenther, Katja Aschenbrenner, Ivonne Stamm, Astrid Bethe, Torsten Semmler, Annegret Stubbe, Michael Stubbe, Nyamsuren Batsajkhan, Youri Glupczynski, Lothar H. Wieler, Christa Ewers

**Affiliations:** 1 Institute of Microbiology and Epizootics, Veterinary Faculty, Freie Universität Berlin, Berlin, Germany; 2 Vet Med Labor GmbH, Ludwigsburg, Germany; 3 Department of Zoology, Institute of Biology, Martin Luther Universität Halle-Wittenberg, Halle, Germany; 4 Department of Zoology, National University of Mongolia, Ulan-Bator, Mongolia; 5 National Reference Laboratory for Antimicrobial Resistance in Gram-negative bacteria, Centre Hospitalier Universitaire de Mont-Godinne, Université Catholique de Louvain, Yvoir, Belgium; 6 Institute of Hygiene and Infectious Diseases of Animals, Veterinary Faculty, Justus-Liebig-Universität Giessen, Giessen, Germany; USGS National Wildlife Health Center, United States of America

## Abstract

Frequent contact with human waste and liquid manure from intensive livestock breeding, and the increased loads of antibiotic-resistant bacteria that result, are believed to be responsible for the high carriage rates of ESBL-producing *E. coli* found in birds of prey (raptors) in Central Europe. To test this hypothesis against the influence of avian migration, we initiated a comparative analysis of faecal samples from wild birds found in Saxony-Anhalt in Germany and the Gobi-Desert in Mongolia, regions of dissimilar human and livestock population characteristics and agricultural practices. We sampled a total of 281 wild birds, mostly raptors with primarily north-to-south migration routes. We determined antimicrobial resistance, focusing on ESBL production, and unravelled the phylogenetic and clonal relatedness of identified ESBL-producing *E. coli* isolates using multi-locus sequence typing (MLST) and macrorestriction analyses. Surprisingly, the overall carriage rates (approximately 5%) and the proportion of ESBL-producers among *E. coli* (Germany: 13.8%, Mongolia: 10.8%) were similar in both regions. Whereas *bla*_CTX-M-1_ predominated among German isolates (100%), *bla*_CTX-M-9_ was the most prevalent in Mongolian isolates (75%). We identified sequence types (STs) that are well known in human and veterinary clinical ESBL-producing *E. coli* (ST12, ST117, ST167, ST648) and observed clonal relatedness between a Mongolian avian ESBL-*E. coli* (ST167) and a clinical isolate of the same ST that originated in a hospitalised patient in Europe. Our data suggest the influence of avian migratory species in the transmission of ESBL-producing *E. coli* and challenge the prevailing assumption that reducing human influence alone invariably leads to lower rates of antimicrobial resistance.

## Introduction

Previous studies have demonstrated high carriage rates of Extended-spectrum beta-Lactamase- producing *E. coli* (ESBL-*E. coli*) in faecal excreta of various wild avian hosts, including birds of prey and waterfowl [1]–[4] Most of these studies conducted in Central Europe, a region with high human and livestock densities [5], [6],facilitating interactions between wild birds and human-influenced habitats like urban environments, wastewater treatment facilities, landfills, and land used for intensive agricultural and livestock farming. It has been suggested that such interactions increase the probability for wildlife to acquire antibiotic-resistant bacteria [7], [8]and preliminary evidence shows that birds of prey carry more of these resistant bacteria when they live in an area with intensive livestock production [9]. Certain ESBL-*E. coli* isolates from wild avian hosts belong to phylogenetic lineages that are closely related to those found in human and veterinary clinical settings, presumably explaining the frequent observation of ESBL-*E. coli* in wild birds [10], [11]Thus, an indirect transmission of ESBL-*E. coli* from humans or domestic animals to wild animals, including wild birds, and vice versa, is plausible. However, the frequencies of such events and the routes of transmission are largely unknown, leaving several unanswered questions; namely whether (a) higher detection rates of ESBL-*E. coli* in avian hosts could be related to spatially linked higher human density, and/or whether (b) prolonged shedding of ESBL-*E. coli* in the birds excreta might compensate for infrequent transmission events.

To begin answering these questions, there is a clear need for studies comparing the microbiota of avian hosts in areas with contrasted exposure to human antimicrobial “practice” (use). This study therefore aimed to (i) assess the rate of ESBL-*E. coli* carriage by birds of prey in remote areas compared to those in Central Europe, (ii) characterize these ESBL-*E. coli* genotypically, and ultimately, (iii) provide preliminary data that might help assess the possible role of migrating avian hosts in the spread of ESBL-*E. coli* into remote environments.

The selection of both the sampling areas and the avian species to be sampled was crucial. Beyond enabling factors like legal access to avian samples from remote areas, it was important to select two sampling sites decidedly different in their human and livestock densities and agricultural practices, but still with comparable avian populations; different groups of avian species seem to differ largely in their carriage rates of ESBL-*E. coli* [11].

We therefore chose sampling spots in semi-desert areas of the South Gobi in Mongolia, among the least densely human populated areas in the world. Besides extensive pasture farming of ruminants, Mongolia features relatively low livestock indices for pigs, cattle and poultry due to the absence of industrial animal breeding. However, as overgrazing by free-ranging livestock, including camels, horses, sheep, goats and cattle, has become a problem in some parts of Mongolia, we were careful not to select these areas. Manure spread on the fields is estimated to be of minor relevance as only a marginal area of the country is arable [5], [6], [12]. By contrast, we selected the sampling area in Saxony-Anhalt, Germany, because it represents typical Central European conditions, e.g. high human densities and industrial animal breeding. High livestock indices for pigs, cattle and poultry presumably lead to increased therapeutic use of antimicrobial substances in livestock farming and intensive liquid manuring [5], [6], [12].

The host-side sampling of birds of prey were considered since (i) there is growing evidence that these may carry ESBL-*E. coli* at a high frequency and (ii) in both of the sampling areas some of the same species were present. Furthermore, the German and Mongolian raptor populations were not connected by migration; the main avian migration routes for raptors in both locations are north-south [1]–[4], [13]. Although we sampled different species of raptors in the two areas to reach a minimum acceptable sample size, it should be stressed that raptors demonstrate common feeding behaviours, distinct from other groups of birds.

## Methods

### Ethics Statement

We carried out the sampling of nestlings in Germany and Mongolia during bird ringing and the animals were released afterward in accordance with the Ornithological Council’s guidelines on the use of wild birds in research [14]. We conducted sampling in Mongolia with the approval and in cooperation with the National University of Mongolia in Ulaan-Baatar, Mongolia. Sampling in Germany was performed under the approval of the State Office of Environmental Protection of Saxony-Anhalt, which also granted M. Stubbe the general permission to ring birds.

According to the IUCN Red List of Threatened species, the conservation status of nearly all animals of this study was of “least concern” (LC), with the exceptions of *M. milvus*, *A. monachus* (both nearly threatened; NT) and *F. cherrug* (endangered, EN).

### Sampling of Birds

In spring 2010, while ringing the nestlings of sixteen wild avian species, we obtained cloacal swabs in both Germany and Mongolia; most of the avian hosts sampled were birds of prey. We sampled four non-raptor species in Mongolia, but the isolates originating from these birds were excluded from the calculation of the number of ESBL-*E. coli* (Tab.1).

We carried out sampling in Central Germany, Saxony-Anhalt, in the Northern region of the Harz-mountains, (around the Hakel-Woodland) in an area of 30 km^2^ around N 51°56′24.5″; E 11°13′13.2″ (human density: 116 n/km^2^, livestock densities: cattle/swine 50–100 n/km^2,^ small ruminants 10 n/km^2^, poultry 1.000–2.500 n/km^2^) [12] and at several sampling spots in the South-Mongolian semi-desert and in West Mongolia during the Mongolian-German Biological Expedition 2010 by M. and A. Stubbe (detailed geographic origin: Tab. 1, human density: 1–2 n/km^2^, livestock densities: swine <1 n/km^2^, cattle 1–5 n/km^2^, small ruminants 5–10 n/km^2^, poultry <10 n/km^2^) [12], [13]. We sampled animals once and shipped cloacal swabs (MASTASWAB containing Amies Medium with charcoal, Mast Diagnostics Reinfeld, Germany) to the lab in Berlin.

**Table 1:** Wild bird species sampled and number of *E. coli* and ESBL-producing *E. coli* isolated from respective host species.

Origin	Total number of birdsand avian species	No. of *E. coli*isolated(% avian hosts)	No. ESBL-*E. coli*(% of all *E. coli* )	Strain nameESBL-*E. coli*	Sampling site ofESBL-*E. coli*
Germany	Total 171	65 (38.0)	9 (13.8)		
	13 Black Kites (*Milvus migrans*)	9 (69.2)	2 (22.0)	IMT21743 IMT21823	all within 30 km^2^ aroundN51°56′24.5″, E 11°13′13.2″
	73 Red Kites (*Milvus milvus*)	32 (43.8)	6 (18.8)	IMT21774 IMT21783 IMT21790 IMT21810 IMT21818 IMT21829	
	68 Buzzards (*Buteo buteo*)	15 (22.0)	1 (6.6)	IMT21813	
	2 Sea Eagles (Haliaeetus albicilla)	1 (50.0)	–	–	–
	1 Spotted Eagle (*Aquila pomarina*)	1 (100.0)	–	–	–
	14 Goshawks (Accicepter gentilis)	7 (50.0)	–	–	–
Mongolia	Total 91	37 (40.7)	4 (10.8)		
	19 Black Kites (*Milvus migrans*)	13 (68.4)	1 (7.6)	IMT23464	N 44°24′03.6″, E 105°21′17.9″
	9 Buzzards (Buteo hemilasius)	3 (33.3)	–		
	30 Black Vultures (*Aegypius monachus*)	11 (36.6)	3 (27.3)	IMT21913	N 47°40′22.4″, E 105°56′51.9″
				IMT23462	N 45°48′05.4″, E 107°15′07.9″
				IMT23463	N 45°46′53.6″, E 107°15′23.7″
	4 Steppe Eagles (*Aquila nipalensis*)	2 (50.0)	–	–	–
	1 Golden Eagle (A*quila chrysaetos*)	0 (0)	–	–	–
	1 Short-toed Eagle *(Circaetus gallicus*)	0 (0)	–	–	–
	3 Eurasian Hobbys (*Falco subbuteo*)	0 (0)	–	–	–
	14 Kestrels (Falco tinnunculus)	4 (28.7)	–	–	–
	8 Saker Falcons (*Falco cherrug*)	2 (25.0)	–	–	–
	2 Lesser Kestrels (*Falco naumanni*)	2 (100.0)	–	–	–
	15 Demoiselle Cranes (*Anthropoides virgo*)*	6 (40.0)	1 (16.6)	IMT23465	N 46°41′32.6″, E 106°31′02.0″
	2 Sandpipers (A*ctitis hypoleucos*)*	0 (0)	–	–	–
	1 Nightjar (Caprimulgus europaeus)*	0 (0)	–	–	–
	1 Hoepoe (*Upupa epops*)*	0 (0)	–	–	–

* Non-birds of prey species, not included in the calculations;

- = no ESBL identified.

### Isolation of *E. coli*

Cloacal swabs were streaked out on CHROMagar orientation (with and without 4 µg/ml cefotaxime; Mast Diagnostica, Reinfeld, Germany) and incubated overnight to isolate *E. coli* and to preselect for cefotaxime-resistant *E. coli*. One colony per sample with coliform appearance on CHROMagar was processed further and bacterial species identification was carried out using the automated VITEK®2 system (BioMérieux, Germany).

### Phenotypic Characterization of ESBL-*E. coli*

*E. coli* isolates showing growth on CHROMagar containing cefotaxime were confirmed as ESBL producers using the phenotypic confirmatory test for ESBL production, performed and interpreted according to CLSI guideline M31-A3 [15].[Using the VITEK®2 system (BioMérieux, Germany) minimal inhibitory concentration testing for antimicrobials was performed according to the guidelines given by the CLSI.

### Genotypic Characterization of ESBL-*E. coli*

The genomic make-up of the confirmed ESBL-*E. coli* was characterised using established PCR protocols with amplification and subsequent sequencing for the most prevalent beta lactam resistance genes (*bla*_CTX-M_, *bla*_SHV_, *bla*_TEM_ and *bla*_OXA_) and non-beta-lactam resistance genes (*tet*(A), *tet*(B), *tet*(C), *sul1*, *sul2*, *sul3*, *strA*, *strB*, *aadA1-like*, *aacC4, acc(6′)-Ib qnrA*, *qnrB*, and *qnrS*) [16]–[26] The presence of the *intI1* and *intI2* genes, encoding class 1 and 2 integrases, was also determined by PCR [23].

### MLST and Phylogenetic Grouping by Structure Analysis

Multi-locus sequence type (MLST) determination was carried out as described previously [27], [28]. Gene amplification and sequencing were performed by using primers specified on the *E. coli* MLST web site (University of Cork website, http://mlst.ucc.ie/mlst/mlst/dbs/Ecoli, accessed 2012 November 29th). Sequences were analysed by the software package Ridom SeqSphere 0.9.39 (Ridom website, http://www3.ridom.de/seqsphere, accessed 2012 November 29th) and sequence types were computed automatically. The phylogenetic group of the *E. coli* strains was determined using the software Structure 2.3.X based on the concatenated sequences of the seven housekeeping genes used for MLST (University of Chicago website http://pritch.bsd.uchicago.edu/structure.html, accessed 2012 November 29th).

### Macrorestriction Analyses by Pulsed Field Gel Electrophoresis (PFGE)

To asses possible clonal relatedness of ESBL-producing *E. coli* isolates, macrorestriction analysis was performed as previously described using a CHEF DRIII System (BioRad, Munich, Germany) [27]. PFGE profiles generated by restriction of chromosomal DNA with *Xba*I were compared digitally using BioNumerics software (Version 6.6, Applied Maths, Belgium). Cluster analysis of Dice similarity indices based on the unweighted pair group method with arithmetic mean (UPGMA) was applied to generate dendrograms depicting the relationships among PFGE profiles. Isolates were considered to belong to a group of clonally related strains if the Dice similarity index of the PFGE pattern was ≥85% [29].

## Results and Discussion

We isolated comparable rates of *E. coli* from birds sampled in the two sampling areas; 38% of the German and 41% of the Mongolian birds carried *E. coli* (Tab. 1). We confirmed ESBL-production in 13.8% (n = 9) of the sixty-five German and in 10.8% (n = 4) of the thirty-seven Mongolian *E. coli* isolates. Although we detected an ESBL-*E. coli,* originating from a Demoiselle Crane, the strain was excluded from the calculations as it represented a non-raptor bird, but we have provided the typing results of the strain in the manuscript. All ESBL-*E. coli* from this study originated from different individual raptors in different nests, thus precluding a possible bias caused by inter-sibling transfer in the nest. By including all birds of the study –even those who did not carry *E. coli*, 5.2% of the wild birds from Germany and 4.5% of those from Mongolia carried ESBL-*E. coli*.

The detection rate of ESBL-*E. coli* observed for the German isolates (13.8%) correlates with data from other studies on raptors from Central Europe where detection rates from 10–20% have been observed [11]. Although based on a limited number of studies available on raptors, high carriage rates seem to be present in Europe independently from the origin of the birds, whether from natural preserves or land used for agricultural production [11]. There is no data on the occurrence of ESBL-*E. coli* in raptors from remote areas, but the detection rates of ESBL-*E. coli* for other avian species (Glaucus Winged Gull) were only about 1%. In this regard, the carriage rates of ESBL-*E. coli* detected in this study among Mongolian raptors (10.8%) is surprisingly high. Furthermore, the ESBL-*E. coli* detected by Hernandez et al. (2010) [30] was typed as O25b:H4-ST131-CTX-15, presenting an anthropogenic clinically important zoonotic pathogen and no commensal *E. coli* [31]. The mere detection of antimicrobial resistant commensal *E. coli* in the Mongolian samples, which has been described for wild birds or rodents in remote areas [32], [33] could have been anticipated, but high rates of clinically important multi-resistant ESBL-*E. coli* like ST648 and ST167 have, to the best of our knowledge, not been yet detected in remote areas. As described in detail in the following, large proportions of the Mongolian ESBL-*E. coli* in this study displayed a high similarity to relevant clinical pathogenic strains from Europe, clearly indicating their zoonotic potential. Thus our data underline that the previous finding of O25b:H4-ST131-CTX-15 in a wild bird was not by chance [30], but that besides commensal *E. coli* displaying antimicrobial resistance, clinical zoonotic pathogens have also reached remote areas in significant numbers.

Genotypic characterisation of the isolates revealed that all ESBL-*E. coli* harboured *bla*_CTX-M_ genes, with *bla*_CTX-M-1_ predominating among German (100%) and *bla*_CTX-M-9_ amongst Mongolian isolates (80%) (Tab. 2). *bla*_CTX-M-1_ represents the predominant ESBL type in poultry, pigs and cattle in Europe, but not in human clinical samples where *bla*_CTX-M-1_ counts for only about 7%, as revealed by a meta-analysis recently published by our group [26]. The high prevalence of *bla*_CTX-M-1_ in the German birds suggests transmission from livestock breeding into the environment, and subsequently, to wild birds, rather than from human sources. To our knowledge, clinical data on ESBL-*E. coli* from Mongolia are not available, but as for the rest of Asia, the major type found in the Mongolian birds (*bla*_CTX-M-9_) only plays a minor role in human clinical samples [34]. *bla*_CTX-M-9_ has also been found less frequently in livestock in Asia, underlining the highly complex spread of ESBL-*E. coli* [34].

**Table 2:** Molecular characteristics of ESBL-producing *E. coli* obtained from wild avian hosts according to phylogenetic background and resistance profile.

Strain	Host	Origin	Ances-tralgroup	ST	STC	ESBL type	Non extended spectrum beta-lactam resistancegenes and integron cassettes
IMT21743	Milvus migrans	Ger	A	744	10	*bla*_CTX-M-1_	bla_TEM-1-like,_ tet(B), sul2, strA, strB, aac(6′)-IB-cr, integron class I
IMT21774	Milvus milvus	Ger	A	744	10	*bla*_CTX-M-1_	bla_TEM-1-like,_ tet(B), sul2, strA, strB, aac(6′)-IB-cr, integron class I
IMT21783	Milvus milvus	Ger	A	744	10	*bla*_CTX-M-1_	bla_TEM-1-like,_ tet(B), sul2, strA, strB, aac(6′)-IB-cr, integron class I
IMT21790	Milvus milvus	Ger	B2	12	12	*bla*_CTX-M-1_	*bla*_TEM-1-like,_ *tet*(A), integron class I
IMT21810	Milvus milvus	Ger	B1	847	none	*bla*_CTX-M-1_	*bla*_TEM-1-like_, *bla*_OXA-1_, *tet*(A), *sul2, strA, strB,* integron class I
IMT21813	Buteo buteo	Ger	A	744	10	*bla*_CTX-M-1_	bla_TEM-1-like,_ tet(B), sul2, strA, strB, aac(6′)-IB-cr, integron class I
IMT21818	Milvus milvus	Ger	A	2199	155	*bla*_CTX-M-1_	tet(B), sul2, strA, strB, integron class I
IMT21823	Milvus migrans	Ger	D	2198	none	*bla*_CTX-M-1_	*bla*_TEM-1-like_, *sul2, strA, strB,* integron class I
IMT21829	Milvus milvus	Ger	AxB1	1640	350	*bla*_CTX-M-1_	sul2, strA, strB, aac(6′)-IB-cr, integron class I
IMT21913	Aegypius monachus	Mon	ABD	117	117	*bla*_CTX-M-55_	bla_TEM-1-like,_ sul2, strA, strB, aac(3)-IV
IMT23462	Aegypius monachus	Mon	A	167	10	*bla*_CTX-M-9_	*sul2, strA, strB*, integron class I
IMT23463	Aegypius monachus	Mon	ABD	648	648	*bla*_CTX-M-9_	bla_TEM-1-like_, bla_OXA-1_, tet(A), sul2, strA,strB, aac (6′)-IB-cr, integron class I
IMT23464	Milvus migrans	Mon	ABD	648	648	*bla*_CTX-M-9_	bla_TEM-1-like_, bla_OXA-1_, tet(A), sul2,strA, strB, aac (6′)-IB-cr, integron class I
IMT23465	Anthropoides virgo*	Mon	B2	2346	none	*bla*_CTX-M-9_	*bla*_TEM-1-like_, *bla*_OXA-1_, *tet*(A), *sul1,* integron class I

Abbreviations: ST = sequence type; STC = ST complex; Ger = Germany; Mon = Mongolia,

* Non-birds of prey species.

Moreover, we detected several other resistance determinants for both sampling areas along with the beta-lactamase encoding genes (Tab. 2); the phenotypic resistance patterns these strains displayed confirmed these results (Tab. S1). Besides the production of ESBLs we also found concomitant resistance to tetracycline, sulphonamide/trimethoprim, and, to a lesser extent, fluoroquinolones. These results confirm recent data on common resistance in wild birds [35]. Ancestral group B2 strains (Sequence type ST12, ST2346), which are believed to be of high extra-intestinal virulence (ExPEC; extra-intestinal pathogenic *E. coli*), were present in one bird from Mongolia and one from Germany. Hybrid group ABD ESBL-*E. coli* strains (ST117, ST648) which are also believed to be of extra-intestinal virulence, predominated in Mongolian birds (60%). Thus several isolates from both sampling areas combined multiresistance with a certain extra-intestinal virulence potential resembling a trend that has been observed in ESBL isolates worldwide and is highlighted by the intercontinental spread of O25b:H4-ST131-CTX-15 which represents an ESBL-producing ExPEC [31].

**Figure 1:**
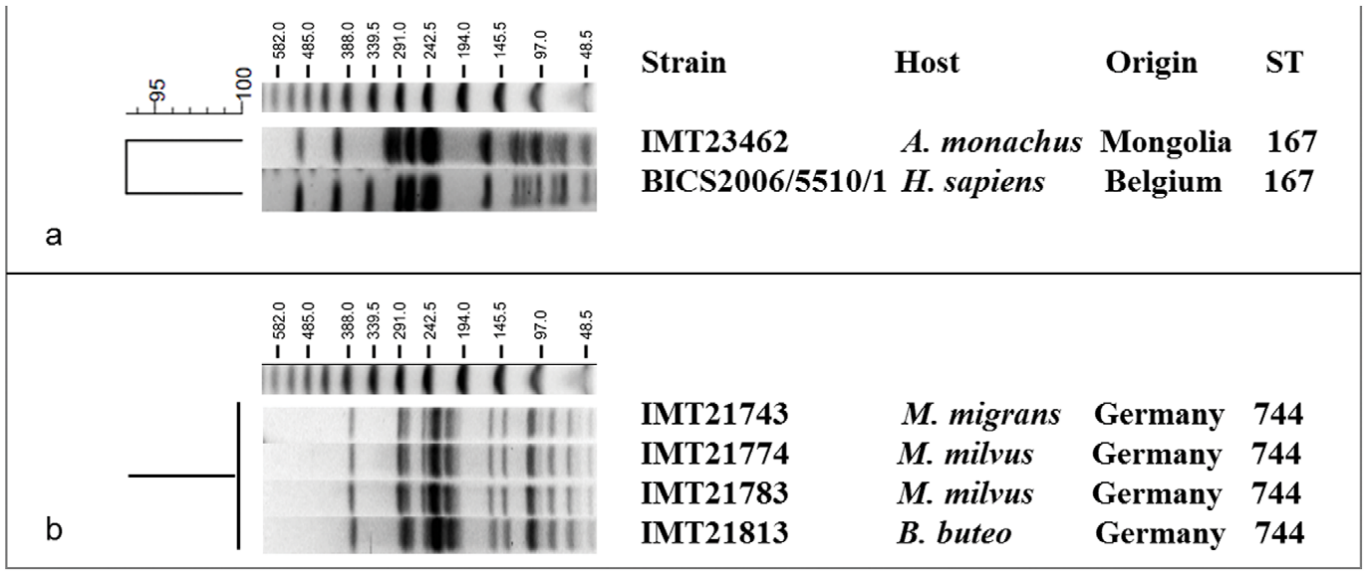
Dendrogram showing (A) the relationship of one avian ESBL-*E. coli* isolate and a human clinical isolate [**39**], both of ST167, and (B) PFGE profiles of four avian ST744 ESBL-*E. coli* isolates based on *Xba*I restriction calculated with Bionumerics 6.6 (Applied Maths, Belgium). ST = sequence type; *A*. = *Aegypius; B*. = *Buteo*; *H*. = *Homo*; *M.* = *Milvus,* A size marker (Lambda Ladder PFG Marker; New England Biolabs GmbH, Frankfurt a. M., Germany) with respective fragment sizes (kb) is given on top of the agarose gel.

Overall, ten different STs were detected among the avian ESBL-*E. coli*; several of these, including ST12 (ancestral group B2), ST847 (B1), ST167 (A), and ST117 (ABD) have already been reported from human and veterinary clinical ESBL-producing isolates [34], [36]–[39]. Interestingly, ST167 belongs to STs that have been associated with the global carriage of ESBL-*E. coli* in humans [34], [38]–[41]. Recently, we found that this phylogenetic lineage was highly prevalent in ESBL-*E. coli* from companion animals (own unpublished data), while to the best of our knowledge, this is the first report of ST167 ESBL-*E. coli* in wildlife. In a previously published study on the epidemiology of ESBL-producing *Enterobacteriaceae* in Belgian hospitals [37] an ESBL-producing ST167 *E. coli* isolate (BICS2006/5510/1) was detected in a clinical sample from a 67-yr old patient with urinary tract infection. Indeed, a comparative macrorestriction analysis of this strain with the Mongolian ST167 Black vulture ESBL-*E. coli* isolate IMT23462 confirmed the clonal relatedness of both isolates (dice similarity index >90%) (Fig. 1 A). This finding is in line with recent data indicating that wildlife principally carries strains from the same phylogenetic background as clinical strains or even identical ESBL strains [11].

Interestingly, four of the isolates collected from wild birds in Germany were all assigned to ST744 and were subsequently found to belong to a single and identical PFGE clone (Fig. 1 B), although they originated from individuals (belonging to three avian species) sampled at different locations within an area of 30 square kilometres. A possible explanation for this could be either the existence of one single environmental origin or a yet unidentified non-point source, thus implying the general occurrence of this particular clone in that area to that time point.

The low-level prevalence of ESBL-*E. coli* that has been reported previously in wild birds from remote areas [30] contrasts with the findings in the present study. This is surprising since it has been recently shown that proximity to human-influenced settings was associated with an increase in antimicrobial-resistant bacteria [42]. The occurrence of ESBL-*E. coli* seems to be species dependent and this may have influenced the high rates obtained in this study [11]. Nevertheless the southward migration of Mongolian Birds to areas with higher human influence and the possible spill-over of multi-resistant bacteria from a spatially segregated, polluted environment might be the reason for the frequent occurrence of ESBL-*E. coli* in the avian hosts in remote areas. This would also contradict previous assumptions that multi-resistant bacterial contaminations should be low in remote environments that lack constant antibiotic pressure [43].

### Conclusions

The possible contribution of avian migration to the transmission of multi-resistant bacteria has been discussed previously [11], [44]. In this study, we found that ESBL-*E. coli* from wild birds originating from Germany and from remote regions of Mongolia differed in their resistance genes and phylogenetic background. This is not unexpected, since the examined avian species do not migrate between Mongolia and Central Europe. Nonetheless, all Mongolian avian hosts sampled in this study undergo southward migration, namely on the Korean Peninsula (Black vulture), to China (Buzzards), and to India (Demoiselle Crane), connecting remote areas to the globalised world with high frequencies of ESBL-*E. coli* in human and livestock [13], [45]–[47].

Although we acknowledge that the presented data are based on a limited number of samples, they clearly suggest the equation “no man, no resistance” is an over simplification [48], as clinically relevant ESBL-*E. coli* are present in remote environments as well. The contribution of international human travel to the spread of multi-resistant *E. coli* has only recently garnered attentions [49] and avian migration follows essentially the same principles. Its importance is often neglected although the number of migrating birds worldwide has been estimated to be five billion a year [50]. We would encourage additional studies focusing on carriage rates, persistence and duration of shedding/excretion of ESBL-*E. coli* in migratory birds on a larger scale. Studies on the necessary frequency of antibiotic exposure to generate significant resistance in wildlife are needed. Such data could help to estimate the potential influence of avian hosts on the pandemic spread of ESBL-*E. coli* into the environment, the community and ultimately, human and veterinary clinical settings.

## Supporting Information

Table S1 **Results of minimal inhibitory concentration testing of avian ESBL-producing** ***E. coli*** **(mg/L).**
(DOC)
