# Supplementary material for: Comparable High Rates of Extended-Spectrum-Beta-Lactamase-Producing Escherichia coli in Birds of Prey from Germany and Mongolia
Source: PLoS One. 2012 Dec 31;7(12):e53039. doi: 10.1371/journal.pone.0053039 (PMC3534101; doi:10.1371/journal.pone.0053039)
Supplement: Table S1 — Results of minimal inhibitory concentration testing of avian ESBL-producing E. coli (mg/L). (DOC) [file pone.0053039.s001.doc]

**Supplementary Table 1: Results of minimal inhibitory concentration testing of avian ESBL-producing *E. coli* (mg/L)**

|  |  |  | **β-Lactams** | | | | | | **Others** | | | | | | |
| --- | --- | --- | --- | --- | --- | --- | --- | --- | --- | --- | --- | --- | --- | --- | --- |
| **Strain designation** | **Host** | **Country** | **Ampicillin** | **Amoxicillin/ Clavulanic acid** | **Cefalexin** | **Cefpodoxim** | **Ceftiofur** | **Imipenem** | **Enrofloxacin** | **Marbofloxacin** | **Chloramphenicol** | **Gentamicin** | **Nitrofurantoin** | **Tetracycline** | **Trimethoprim/ Sulfamethoxazole** |
| IMT21743 | *Milvus migrans* | Germany | >=32 | 4 | >=64 | >=8 | >=8 | <=1 | >=4 | >=4 | 4 | 2 | <=16 | >=16 | >=320 |
| IMT21774 | *Milvus milvus* | Germany | >=32 | 4 | >=64 | >=8 | >=8 | <=1 | >=4 | >=4 | 4 | 2 | <=16 | >=16 | >=320 |
| IMT21783 | *Milvus milvus* | Germany | >=32 | 4 | >=64 | >=8 | >=8 | <=1 | >=4 | >=4 | 4 | 2 | <=16 | >=16 | >=320 |
| IMT21790 | *Milvus milvus* | Germany | >=32 | 4 | >=64 | >=8 | >=8 | <=1 | <=0.12 | <=0.5 | 4 | <=1 | <=16 | >=16 | <=20 |
| IMT21810 | *Milvus milvus* | Germany | >=32 | 4 | >=64 | >=8 | >=8 | <=1 | <=0.12 | <=0.5 | 4 | <=1 | <=16 | >=16 | >=320 |
| IMT21813 | *Buteo buteo* | Germany | >=32 | 4 | >=64 | >=8 | >=8 | <=1 | <=0.12 | <=0.5 | 4 | <=1 | 64 | >=16 | >=320 |
| IMT21818 | *Milvus milvus* | Germany | >=32 | 4 | >=64 | >=8 | >=8 | <=1 | <=0.12 | <=0.5 | 4 | <=1 | 64 | >=16 | >=320 |
| IMT21823 | *Milvus migrans* | Germany | >=32 | 4 | >=64 | >=8 | >=8 | <=1 | <=0.12 | <=0.5 | 4 | 2 | 64 | <=1 | >=320 |
| IMT21829 | *Milvus milvus* | Germany | >=32 | 4 | >=64 | >=8 | >=8 | <=1 | <=0.12 | <=0.5 | 4 | 2 | <=16 | <=1 | >=320 |
| IMT21913 | *Aegypius monachus* | Mongolia | >=32 | 4 | >=64 | >=8 | >=8 | <=1 | 1 | 1 | >=64 | >=16 | <=16 | >=16 | >=320 |
| IMT23462 | *Aegypius monachus* | Mongolia | >=32 | 4 | >=64 | >=8 | >=8 | <=1 | >=4 | >=4 | 8 | <=1 | 64 | <=1 | <=20 |
| IMT23463 | *Aegypius monachus* | Mongolia | >=32 | 4 | >=64 | >=8 | >=8 | <=1 | >=4 | >=4 | 16 | <=1 | 32 | >=16 | >=320 |
| IMT23464 | *Milvus migrans* | Mongolia | >=32 | 4 | >=64 | >=8 | >=8 | <=1 | >=4 | >=4 | 16 | <=1 | <=16 | >=16 | >=320 |
| IMT23465 | *Anthropoides virgo** | Mongolia | >=32 | 4 | >=64 | >=8 | >=8 | <=1 | 1 | 1 | 4 | >=16 | <=16 | >=16 | >=320 |
